# Optimization of IL-1RA structure to achieve a smaller protein with a higher affinity to its receptor

**DOI:** 10.1038/s41598-022-11100-3

**Published:** 2022-05-06

**Authors:** Mahsa Nouri Barkestani, Sina Naserian, Fatemeh Khoddam, Sara Shamdani, Bijan Bambai

**Affiliations:** 1 Department of Systems Biotechnology, National Institute of Genetic Engineering and Biotechnology (NIGEB), Tehran, Iran; 2 INSERM UMR-S-MD 1197, Hôpital Paul Brousse-Bâtiment Lavoisier, 12-14 avenue Paul Vaillant Couturier, 94800 Villejuif, France; 3 Paris-Saclay University, Villejuif, France; 4 CellMedEx, Saint Maur Des Fossés, France

**Keywords:** Proteins, Intracellular signalling peptides and proteins, Computational biology and bioinformatics

## Abstract

Interleukine-1 family cytokines are key orchestrators of innate and adaptive immunity. In particular, up-regulation of IL-1R1 via its agonistic ligands consisting of IL-1β and IL-1α is implicated in a variety of human diseases, such as rheumatoid arthritis, psoriasis, type I diabetes, amyotrophic lateral sclerosis, and dry-eye disease. Until now, there are no small-molecule inhibitors of the IL-1R1 with increased antagonistic potency to be used for the treatment of peripheral inflammation. The objective of this study was to engineer a low-molecular-weight version of IL-1RA with increased affinity and enhanced antagonistic activity for potential therapeutic use. To develop a smaller protein–ligand with a better affinity to IL-1R, we used bioinformatics studies and in silico simulations to anticipate non-binding areas on IL-1RA. In this study, we have identified a 41aa (F57-F98) non-binding site of IL-1RA. Overall RMSF of the Truncated complex (1.5 nm) was lower than the Native complex (2 nm), which could prove higher stability of the Truncated complex. The free binding energy of the T-IL-1RA (− 1087.037 kJ/mol) was significantly lower than the IL-1RA (− 836.819 kJ/mol) which could demonstrate a higher binding affinity of the truncated ligand with its receptor as a result of new important interactions. These findings have demonstrated a higher binding affinity of the T-IL-1RA with its receptor than the native protein. These results should**:** have an impact on the development of new treatments that block IL-1 signaling, although more research is needed in vitro and in vivo.

## Introduction

Interleukine-1 (IL-1) is one of the first known interleukins involved in several immune responses^1,2^. IL-1 family consists of 11 members: IL-1β, IL-1α, IL-18, IL-33, IL-1F5 to IL-1F10, and IL-1 receptor antagonist (IL-1RA)^3^. Overproduction of IL-1β and IL-1α and consequently the up-regulation of IL-1R1 has been implicated in numerous chronic inflammatory and auto-immune disorders^4^. IL-1RA, IL-1β, and IL-1α are composed of 12 β-strands and the linkers between them which form an anti-parallel β-barrel with a size of ~ 17 kDa^5^. IL-1β and IL-1α exert pro-inflammatory effects through initially binding to the IL-1R1^6^, ligand-recognition receptor subunit, which recruits a signaling subunit receptor termed Interleukin-1 Receptor Accessory Protein (IL-1RAcP)^7,8^. Ectodomains of their receptors consisted of three immunoglobulin-like domains^9^. The juxtaposition of the intracellular Toll–IL-1 receptor domains of two subunit receptors after binding to agonistic ligands triggers intracellular signaling, which leads to activation of the nuclear factor κB (NF-κB) and mitogen-activated protein kinase (MAPK) pathways, therefore, a multitude of inflammatory mediators, such as cytokines and chemokines are expressed^10,11^.

The pro-inflammatory activities of these cytokines can be tightly regulated through pathways that include both extracellularly and intracellularly levels. Naturally occurring inhibitors include protein receptor antagonist IL-1RA, decoy receptor IL-1R2^12^, and soluble forms of all IL-1 receptors^13,14,15,16^. Firstly, IL-1RA competitively binds to the IL-1R1 with a high affinity to prevent its binding with agonistic ligands. This complex is not able to recruit the accessory protein subunit (signaling subunit), therefore no signal transduction occurs^4,8^.

Currently, a great number of anti-inflammatory drugs are actively used to inhibit the signal cascade of IL-1R1 to cure a broad spectrum of inflammatory diseases^17^. Rilonacept is a dimeric chimer protein consisting of Ig-like domains of IL-1R1 and IL-1RAcP along with the Fc-fragment of human IgG. It can capture the IL-1β and IL-1α proteins and inhibit their function^18,19^. Canakinumab is the human IL-1β monoclonal antibody that binds to human IL-1β with an IC_50_ of about 43 pM^20^. Anakinra is a non-glycosylated recombinant version of IL-1RA by the presence of an additional N-methionine which competitively binds to IL-1R1 and blocks its actions with an IC50 around 1.6 nM^21^. They have already been approved for the treatment of autoinflammatory disorders^22^, such as rheumatoid arthritis^23^, type 2 diabetes mellitus^24^, systemic-onset juvenile idiopathic arthritis^25^, osteoarthritis^26^, and adult-onset Still’s disease^27^**.** Despite the high efficacy of available drugs, they have some disadvantages, such as low receptor affinity and efficacy of Anakinra, which results in the need for daily injection at very high concentrations, which gives rise to toxic systemic effects, risk of infection, and neutropenia. Moreover, the production of antibody form drugs such as Canakinumab and Rilonacept is expensive and not possible in the bacterial system^28^.

Recently, a wide range of studies for designing a lower molecular mass IL-1R1 antagonists have been performed. In 1996, Yanofsky et al. represented the possibility of a small molecule antagonist with high affinity which binds to IL-1R1 with IC50 of 2.6 nM. Although these investigations bring forward a high-affinity small molecule antagonist, the disadvantages are still similar to other protein drugs such as Anakinra: the molecular weight is still high and the method of administration is limited to the injection^29,30^. In this project, we focused on increasing the receptor affinity and therefore, protein efficacy, thus a low-molecular-weight IL-1RA with improved functional activity and receptor affinity could be expected for the aim of peripheral inflammation treatment.

Protein engineering technology is capable of generating macromolecules with enhanced therapeutic efficacy^31^. Identifying the contact regions between a ligand and its binding receptor is essential for creating new therapeutic proteins that block the interaction^32^. Unfortunately, the large ligand-receptor interface and hidden contact regions inside the binding interface of ligand-receptor pose a challenge in recognizing binding sites for low-molecular-weight antagonist development^33^. Therefore, it is not feasible to obtain the binding mode of protein complexes by experimental methods alone^34^. MD simulations have been widely applied in exploring conformational space, accurate binding modes and binding ability, protein folding, dynamic structural transformation processes, and binding energy information^35^, which have been proven to be valuable for the discovery and design of small-molecules targeting ligand-receptor interface^36,37^. Here, we utilized bioinformatics tests and in silico simulations to predict non-binding regions on IL-1RA to design a smaller protein–ligand with a higher affinity to IL-1R. We kept interactive sites of the ligand with the IL-1RI subunit and truncated the protein from non-binding sites, without altering three-dimensional (3D) structures of IL-1RA.

## Methods

### Protein selection

**Figure 1:**
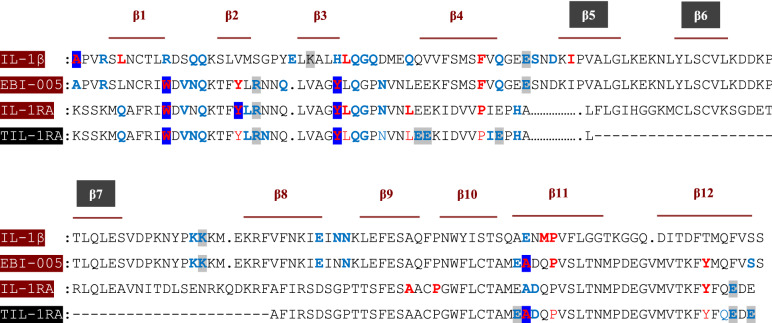
Identification and characterization of a novel truncated IL-1RA that shares homology with IL-1β and EBI-005. Sequence alignment of ligands with colorful residues implicated in interaction with IL-1R1, identified by ZDOCK within 4 Å. Hydrophobic interaction: red, Hydrogen bond: blue and Ionic interaction: gray.

In this study for predicting the crucial binding sites and conserved sequences of IL-1RA, we have used other IL-1 ligands. The binding sites of IL-1β and EBI-005 (chimer protein derived from IL-1RA and IL-1β) were more similar and overlapped to the binding sites of IL-1RA in complex with IL-1R1, therefore we have selected these two ligands and omitted others, for showing the further results to avoid redundancy. Protein sequence alignments could identify regions of similarity that may reflect biological relationships among the input sequences. Here we used the protein BLAST tool provided by NCBI for running BLAST of ligands for comparing protein query sequence against a protein sequence subject^38^ (Fig. 1). Ligand-receptor docking simulations were performed by Z-DOCK^39^ to investigate the inter-protein interaction of ligands in complex with their competitive receptor IL-1R1, which delineated crucial overlapped β-sheets of three ligands involved and not involved in the interaction. The interacting residues are highlighted in Fig. 1. Based on binding site similarity IL-1RA, IL-1β and EBI-005 were selected.

Crystal structures of selected ligands in complex with IL-1R1 receptor, presented in RCSB Brookhaven Protein Data Bank (PDB) were chosen with entry codes: 1ILR(IL-1RA), 1ITB (IL-1β), and 4GAI(EBI-005), respectively with 2.1 Å resolution/152 amino acids, 2.5 Å resolution/152 amino acids and 1.49 Å resolution/153amino acids.

### Identifying truncating residues

**Figure 2:**
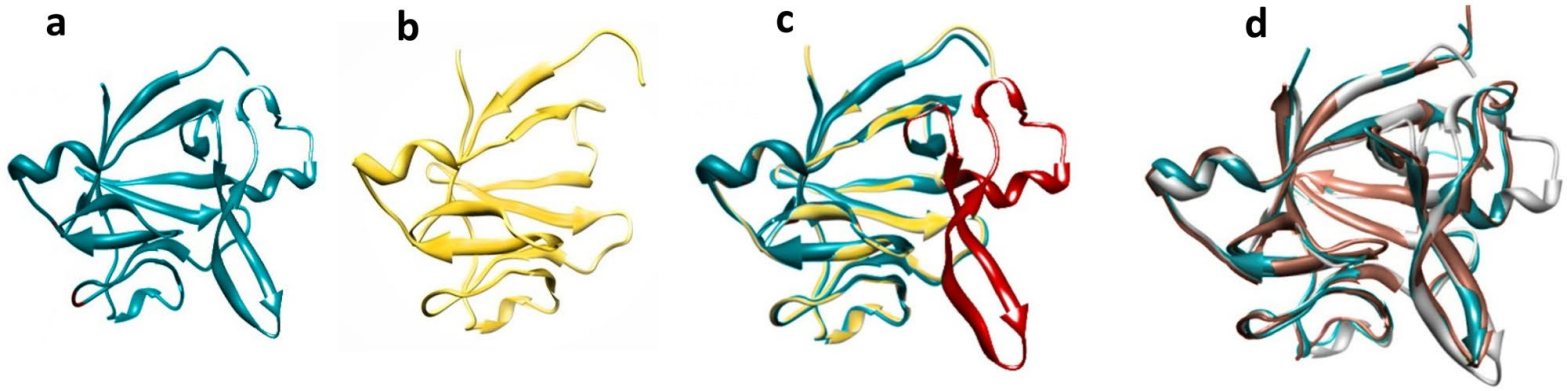
3D structure of IL-1 ligands. (**a**) 3D structure of IL-1RA in comparison with T-IL-1RA model (**b**). (**c**) Superimposition of IL-1RA and T-IL-1RA (superimposed RMSD 0.407 Å). (**d**) Superimposition of IL-1RA (blue ribbon), IL-1β (pink ribbon), and EBI-005 (white ribbon) (superimposed RMSD 0.407 Å).

Protein 3D-superimposition was performed using chimera software 25.42.1611^40^ to identify similarities of protein folds (Fig. 2c,d). Based on previous results, IL-1RA, IL-1β, and EBI-005 exhibit scant identity in sequence (Fig. 1) despite the high similarity in a three-dimensional structure predicted by protein superimposition. Selected ligands are composed of 12 anti-parallel β-strands, adopting a conserved signature β-trefoil fold. They bind the same receptor (IL-1R1) through overlapped β-sheets. The receptor-binding site can be subdivided into site A and B which site A is more affine for IL-1RA while the IL-1β bind receptor with higher affinity in site B. In this project, we have shown the binding and non-binding sites of three ligands in the complex with IL-1R1 to predict the proper truncating site (Fig. 1).

### Truncated IL-1RA protein preparation

**Figure 3:**
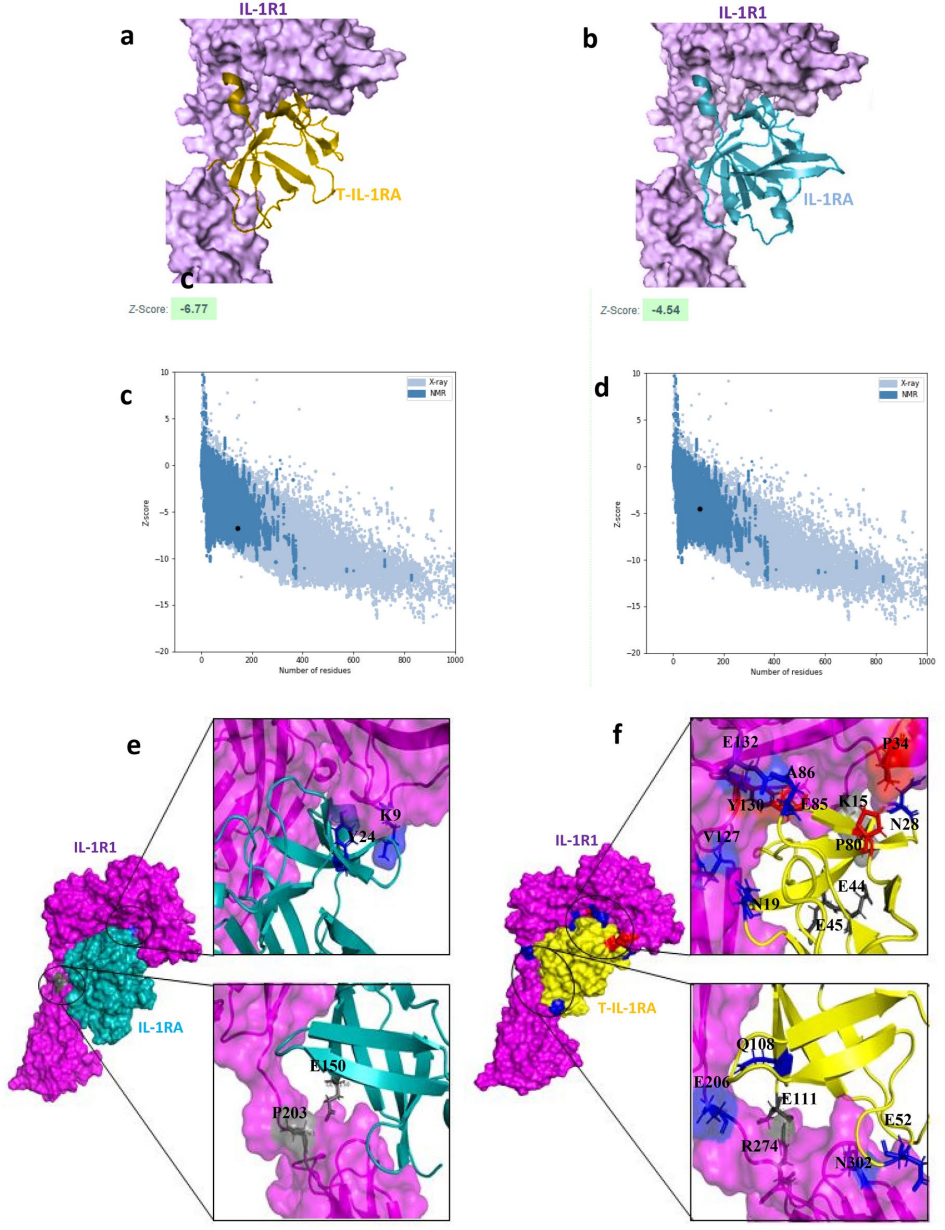
3D structure comparison of IL-1RA and T-IL-1RA in complex with their receptor IL-1R1. (**a**) 3D structure of IL-1RA in complex with IL-1R1. (**b**) 3D structure of T-IL-1RA in complex with IL-1R1. (**c**) Structure model validation of native and (**d**) Truncated ligands, using ZDOCK server. (**e**) Residues of native ligand involved in interaction with IL-1R1 which do not make contact with receptor in truncated ligand/receptor complex. (**f**) New residues of Truncated ligand involved in interaction with IL-1R1 which do not make contact with receptor in Native ligand/receptor complex. Specific interactions of IL-1RA/IL-1R1 Hydrophobic interaction (red), Hydrogen bond (blue), and Ionic interaction (black).

Random truncation was performed on specifically selected low-affinity sites of IL-1RA (Supplementary file 1), afterward, homology models of the truncated proteins were constructed using the automated homology modeling software MODELLER6v2^41^. According to structural similarity to the natural IL-1RA, 100 models were selected for the docking (Supplementary file 2). The molecular docking was performed by the Z-DOCK program which applies a fast Fourier transform to find all feasible binding modes of proteins^42^. For each model, the top 2,000 predictions are given to the RosettaDock program to eliminate clashes and improve energies^43^, and then the ZRANK program re-ranks all models. Visual analysis of the interactions between models and receptor were performed in Chimera software^44^. The modes of interactions of Truncated IL-1RA are displayed in Fig. 3.

### Protein model validation

The PDB file of complexes (IL-1RA/IL-1R1, T-IL-1RA/IL-1R1) was converted to the topology files, adapted for Gromacs package using MDWeb (http://mmb.irbbarcelona.org/MDWeb/) server. Molecular Dynamic (MD) simulations were conducted in GROMACS 4.5.5^45^ using OPLS-AA all-atom force field and SPC216 water model implemented on Intel Xeon 2 × 6-Core W3530 2.8 8 M 1366 Processor with Bio-LINUX 8 operation system. MD simulation was carried out in a dodecahedron box (> 1.2 nm between the protein edge and the box), filled by SPC216 water molecules. According to the native IL-1RA crystallography structure, standard protonation states of the residues were used and the charges of the system were neutralized by replacing water molecules with Na + and Cl^-^ ions^46^. The energy of the system was minimized using the steepest descent algorithm followed by the conjugate gradient procedure, afterwards, all bonds were constrained by the LINCS algorithm. The temperature coupling was carried out using a modified Berendsen thermostat in a coupling time constant, of 0.1 ps. The pressure of the system was held around 1 bar using the Parrinello-Rahman barostat method with a coupling constant, of 0.1 ps. Bond lengths were constrained using the LINCS algorithm in 2 fs time steps. Terminally, MD simulations were performed at 310 K, according to natural biological temperature which our protein is functional, for 50 ns^47^. In all simulations first 10 ns were ignored and the analysis starts from 10 to 50 ns. PyMOL, Chimera, MM-PBSA, and XMGrace software were used to analyze and prepare publication-quality figures^46^.

### RMSD and RMSF calculation

The backbone root means square deviation (rmsd), which indicates protein structure stability, is a crucial analysis to evaluate the MD simulations. Backbone RMSD was calculated using the Gromacs package included tool, g_rms. Adapted crystal structure used as a reference, time to reach stable RMSD was indicated and the first unstable stage was discarded for more analysis to ensure that calculated results reflect protein behavior in the given temperature (Fig. 5a). Protein backbone fluctuations were determined by computing RMSF values using the GROMACS package g_rmsf tool. The RMSF value was calculated in the different temperature trajectories for Cα atoms of all residues for the average structure as a reference (Fig. 5b).

### Hydrogen Bonds, Electrostatic interactions and Salt Bridges

The g_hbond tool was utilized to compute the total number of protein–protein and protein-solvent hydrogen bonds. The g_hbond calculates the number of donor–acceptor pairs with appropriate angle and distance cutoff for hydrogen bond formation. The angle cutoff (angle formed by the hydrogen, donor, and acceptor atoms) has been set at 60° and the distance cutoff has been set to 0.35 nm. The electrostatic interactions have been computed by calculating the distances between all negatively charged groups and all positively charged groups in the trajectories. Salt bridges in trajectories were calculated between oppositely charged residues by the Visual Molecular Dynamics (VMD) program. Salt bridge cut-off length was set in 0.4 nm distance and persistence for at least 20% of the frames (Table 1).

**Table 1:** Decomposition of the IL-1RA/IL-1R1 and T-IL-1RA/IL-1R1 energies by interaction type (kJ/mol).

	Van der waals	Electrostatic	Polar solvation	SASA	Total
Native complex	− 791.118 (53.439)	− 1176.423 (158.613)	1233.940 (126.524)	− 103.219 (5.149)	− 836.819 (125.115)
Truncated complex	− 785.011 (53.511)	− 2214.386 (248.189)	2023.099 (225.820)	− 110.739 (5.672)	− 1087.037 (120.202)

## Results

### Creating truncated-IL-1RA.

Here we utilized the protein BLAST tool provided by NCBI for running BLAST of ligands and residues implicated in protein interaction were determined by ZDOCK. This information led to the recognition of the identical low-affinity site at all three ligands (IL-1RA/IL-1β/EBI-005). Subsequently, we mapped the deletion cluster of IL-1RA, around residue 50–100 (β5-β6-β7) which has the least implication in ligands-receptor (IL-1R1) binding interface and is far from the core of protein structure (Fig. 1). According to the structural similarity to the native protein and the Z-score, we have selected the T-IL-1RA protein model.

Homology modeling data and superimposition of T-IL-Ra and native protein revealed that after the truncation of β5-β6-β7 (57–98 residues truncated), the other β-sheets preserved their parental structure with superimposed RMSD 0.407 Å (Fig. 1). 3D structures of IL-1 ligands (IL-1RA/IL-1β/EBI-005) show a similar structural fold, i.e., they are constituted by a 12-stranded beta-trefoil domain with the linkers between them^10,48^. These ligands share only 22% sequence identity, but they are structural homologs^49^. Based on this information we hypothesized that the IL-1 family ligands could be flexible for sequence alteration, preserving their overall conformation. This idea encouraged us to design a series of truncated-IL-1RA that preserve parental structure and contact regions to the receptor (IL-1R1). Structural superimposition of IL-Ra, IL-1β, and EBI-005 reveals several similarities that may account for the ligand-receptor binding sites (Fig. 2c,d).

### T-IL-1RA/IL-1R1 binding interface in comparison with IL-1RA/IL-1R1

As mentioned, the deletion clusters in the β5-β6-β7 strands, suggest that this area is favorable for truncation. It is evident in the structure that this area is located far from the core of protein structure and it does not intensively participate in the interaction with IL-1R1. Crystallography data analysis of IL-1RA/IL-1R1complex (Fig. 3a,b) revealed that the interface between IL-1RA and IL-1R1 contains strong contacts between β1–β2, β2–β3, β3–β4, β10–β11 loops of ligand and D1D2 domain of the receptor, where residues W16, Y24, Y34, L35, L42, Y147 from IL-1RA formed hydrophobic interaction with K111, K9, P123, F108, L112 of IL-1R1 and R26, E150 had ionic interaction with I10, P203 residues of IL-1R1. The Z-score for T-IL-1RA and IL-1RA were predicted to be − 6.77 and − 4.54, respectively by the ProsaWeb server (Fig. 3c,d). The higher negative score of T-IL-1RA than the wild-type protein ensures the maximum quality of the modeled truncated protein.

As it is demonstrated in Fig. 1, the binding interface of the engineered ligand is significantly similar to the native protein, besides a few extra interactions made in the interface of the T-IL-1RA/IL-1R1 complex (Fig. 3e,f). The new residues of T-IL-1RA involved in interaction include A86, P89 which formed hydrophobic interaction with Y130, P34 of IL-1R1, N10, N28, E52, E85, Q108 had hydrogen bond with v127, K15, N302, E132, E206 residues of IL-1R1 and E44, E45, E111 had ionic interaction with K15, R274 residues of IL-1R1.

### T-IL-1RA antagonistic feature survey via comparing of IL-1/IL-1R1/IL-1RAcP complexes

**Figure 4:**
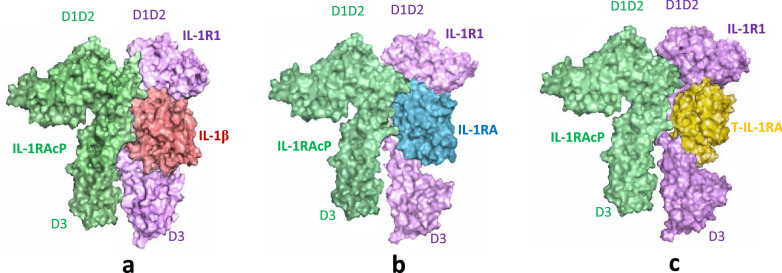
Comparison of natural and truncated IL-1 ligand in the complexes with IL-1R1/IL-1RAcP (**a**) surface representation of IL-1β/IL-1R1/IL-1RAcP structure. (**b**) Surface representation of IL-1RA/IL-1R1 in complex with IL-1RAcP. (**c**) Surface representation of T-IL-1RA in complex with T-IL-1RA.

Previous studies have introduced two chimeras derived from IL-1RA/IL-1β, differ just in 23 residues which are fully antagonist and agonist ligands. Based on chimera construction and crystal structure analysis the crucial residues that determine agonism vs. antagonism are KGGQ/138–141, I143, and D145 on IL-1β^50^. In this study, for the prevention of turning the antagonist into the agonist, we have kept this region intact. The crystallography structure studies of ligands in the complex with not only recognition receptor (IL-1RI (A001241)), but also co-receptor (IL-1RAcP (A003536)) suggested low affinity of IL-1RA to the IL-1R1-D3 domain in the conformational basis of the antagonism. IL-1RAcP is a co-receptor that only can bind to the binary complex of the IL-1R1/IL-1α-β complex^51^. For the stabilization of ternary complex interactions (IL-1/IL-1R1/IL-1RAcP), the D3 domain of IL-1RAcP has to turn around the binary complex to bind the D3 domain of IL-1R1. The D3 domain of IL-1R1 in the complex with IL-1RA stays far away from the D3 domain of IL-1RAcP, which is anticipated to disrupt the D3-D3 binding interface. As it is obvious in our protein model, the binding interface of T-IL-1RA is similar to the parental protein which is important for antagonistic features, therefore the D3 domain of IL-1R1 stays far away from the complex conduces decreased affinity between D3-D3 domains of IL-1R1-IL-1RAcP (Fig. 4).

### Protein structure validation

#### Molecular dynamic simulation

**Figure 5:**
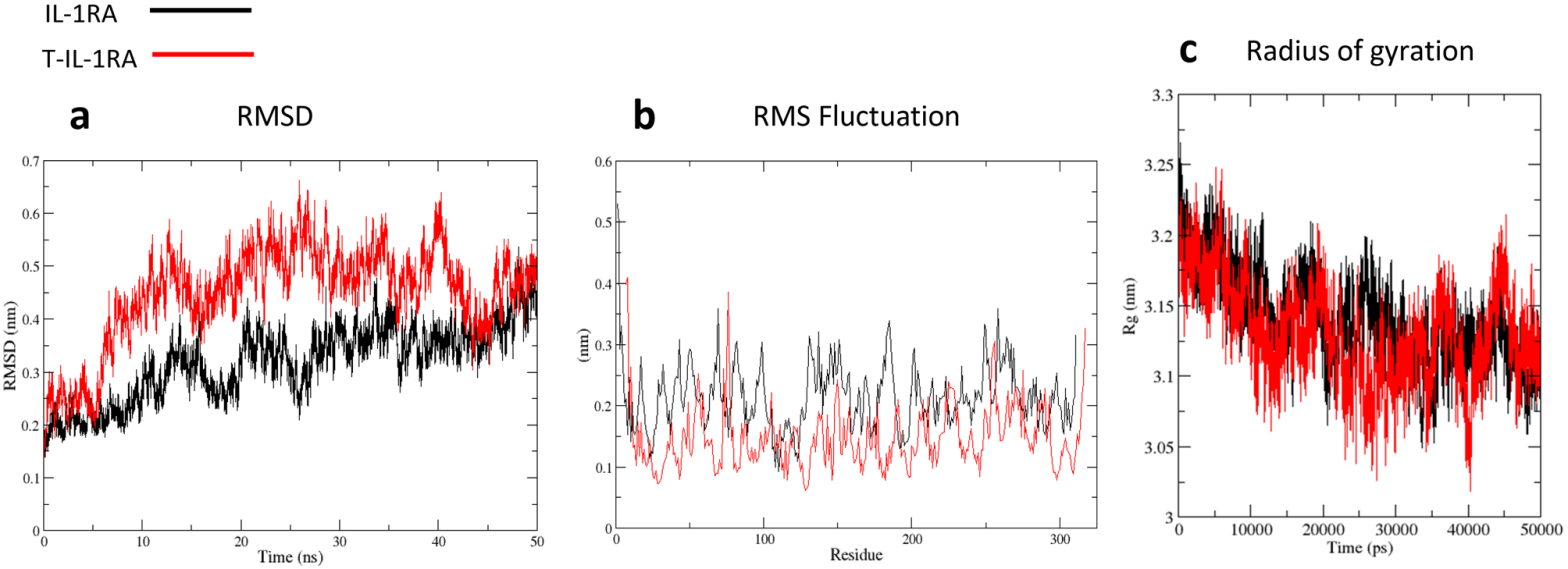
Molecular dynamic output analyses. (**a**) Root-mean-squared deviation plot (**b**) Root-mean-squared fluctuation plot and (**c**) Radius of gyration plot for each system over each 50 ns production run.

To investigate the structural changes in the protein–protein complex induced by ligand binding, several conformational properties were analyzed, such as root mean square deviation (RMSD), root mean square fluctuations (RMSF), the radius of gyration (Rg), number of hydrogen bonds (NHBs), electrostatic interactions and salt bridges. RMSD (nm) vs. time (ns) for all the backbone atoms of IL-1RA/IL-1R1 and T-IL-1RA/IL-1R1 complex simulations were calculated to survey the stability of complexes. As shown in Fig. 5a, early in the simulation of complexes, IL-1R1 domains turn around the ligands because of the flexibility of the linker between the D1D2 and D3 domain, causing an immediate ascent in the overall RMSD value. From 8.5 ns onwards truncated complex attained the approximate equilibrium phase with the RMSD value averaged around 4.6 Å, whereas, the native complex trajectory experienced an ascending trend, which suggested relatively higher stability of T-IL-1RA complex than native complex. Both systems gradually tended to converge in the last 8 ns (Fig. 5a).

RMSF of T-IL-1RA/IL-1R1 and IL-1RA/IL-1R1 complexes were computed to investigate changes in protein flexibility of the complex upon ligand binding. RMSF fluctuation plot of Cα carbon atoms vs time (50 ns) separately for two complexes is shown in Fig. 5b Residues in T-IL-1RA/IL-1R1 complex experienced minor fluctuation and the overall RMSF of the truncated complex was lower than the native complex, which indicated this complex was relatively more stable during the simulation process. New interactions involved in stabilizing the truncated complex could play an important role in minimizing the fluctuations and maintaining the proteins in a rigid structure to simplify the formation of the complex (Fig. 5b).

The radius of gyration is a significant parameter to survey the compactness of protein. The radius of gyration for T-IL-1RA/IL-1R1 and IL-1RA/IL-1R1 complexes showed fluctuation in Rg value until 10 ns, afterward attained virtually stable Rg value. It was indicated that the Rg average values for truncated and native systems were around 3.11 nm and 3.13 nm, respectively. The lower Rg value of truncated ligand bond to the receptor than the native complex can be attributed to the elimination of ligand-space-barrier. Therefore, T-IL-1RA/IL-1R1 complex showed higher compact than the native ligand (Fig. 5c).

#### Interaction energetic feature

MM-GBSA method was used to calculate the binding free energy of systems. The average binding free energies and detailed energetic contribution components of 50 ns were shown in Table 1. Interestingly, the free energy of the truncated system (− 1087.037 kJ/mol) is significantly lower than the native system (− 836.819 kJ/mol) which could demonstrate a higher binding affinity of the truncated ligand with its receptor than the native protein. This result conforms to the outcomes obtained from RMSF analysis. Furthermore, dissecting the binding free energy into contributing components showed that the electrostatic interaction in truncated complex (− 2214.386 kJ/mol) had a major role in the low free energy of the truncated system and the system stability.

## Discussion

The greatest obstacle to target a ligand-receptor interface is buried binding sites and conformational changes of a protein in interacting with different components. Moreover, conventional de novo drug designing is costly, time-consuming, and laborious^52^. Employing *in-silico* approaches have been proven to be beneficial to accelerate the process of protein manipulation and drug development ^53,54^.

A wide range of studies has demonstrated molecular dynamics simulation of proteins as a promising approach not only for the characterization of protein behavior, dynamic, and structure but also a means of assessing accurate binding modes and binding energies of ligand-receptor interactions, which is difficult to obtain by the current experimental methods^37^.

IL-1RA is one of the newest therapeutic targets to block IL-1 activity. Minimizing adverse effects while maintaining the efficiency of current drugs is under development^55^. We suppose that a smaller form of IL-1RA with higher affinity to the receptor would be advantageous in that it would be potentially simpler to administer^56^, could be effective trans-epithelially^57^, be hopefully devoid of adverse effects caused by the need for frequent injections^58^ and be less costly^59^ while having an increased efficacy. The main aim of the current study was to develop a truncated and more effective form of IL-1R1 antagonist for the treatment of peripheral inflammation or any other possible complications.

Considering EBI-005 chimer proteins, alongside a wide range of mutants and peptides derived from natural IL-1 ligands (AF10847) with increased antagonistic potency, we hypothesize conformational flexibility of IL-1R1 to bind different ligands. Moreover, despite the extensive IL-1/IL-1R1 complexes interface, a significant portion of binding energy is generated in a compact interface of the ligand-receptor^60^ the key residues of ligands are discontinuous in the primary sequence though contiguous to each other on the surface of the folded protein. Previously reported high-affinity peptides contained key residues of IL-1RA involved in binding to the IL-1R1, mimics the contact residues and binding mode of IL-1RA. Based on these results, we have suggested that β-trefoil family proteins in the IL-1 display structural stability and flexibility to the sequence alteration by preserving crucial binding sites and protein fold^29^.

Based on these hypotheses and following a wide range of IL-1 ligands-receptor sequence mapping, we identified a stretch of 40–50 amino acids of three targeted ligand (IL-1RA/ IL-1β/ EBI-005) with low primary sequence homology, which has no interaction with IL1R1 and it is far from the protein core (Fig. 1). Therefore, we suggested truncating this site from IL-1RA, would not dramatically alter the secondary as well as tertiary structure of the protein compared with that of wild-type IL-1RA (Fig. 2). To gain insight into the mechanism by which truncated-IL-1RA binds to its receptor, 3D structural models were constructed by homology modeling based on known structures of human IL-1RI and IL-1RA (Fig. 3). We used this structure as the template for our in silico docking and molecular dynamics simulations to examine the interface between engineered protein and its receptor. Unexpectedly, this deletion displayed significantly increased IL-1RA/IL-1R1 affinity (Table 1). For the RMSD value, truncated complex trajectory attained the proximate equilibrium from 8.5 ns onward (Fig. 5a) and RMSF value of native complex was higher than truncated complex (Fig. 5b) which indicated lower fluctuation as a result of new interactions and higher stability of T-IL-1RA/IL-1R1 complex than IL-1RA/IL-1R1. A Higher Rg value of T-IL-1RA/IL-1R1 showed that deleting this site lets IL-1R1 turn more tightly around the ligand (Fig. 5c).

In this study, we proposed the possible critical sites on ligands implicated in interaction with IL-1RAcP to prevent turning antagonist into agonist during protein engineering processes, therefore, the interacting angle of T-IL-1RA, which was similar to the native protein antagonist enabled the binary complex to recruit the signaling subunit IL-1RAcP and retained its antagonistic feature (Fig. 4).

## Conclusions

In conclusion, our in silico simulations resulted in a novel 110–amino acid antagonist of IL-1, that properly binds to all three domains of IL-1RI with higher affinity. We suggest the truncated region may act as a space barrier, therefore elimination of this site lets the D3 domain of the IL-1R1 wrap around the ligand with higher flexibility and encompass the ligand more tightly. These findings should impact the development of new therapeutics that neutralizes IL-1 signaling but it needs to be examined in vitro and in vivo.

## Supplementary Information

Supplementary Information 1.

Supplementary Information 2.

## Data Availability

All data generated or analysed during this study are included in this published article [and its supplementary information files].
