# Supplementary material for: Optimization of IL-1RA structure to achieve a smaller protein with a higher affinity to its receptor
Source: Sci Rep. 2022 May 6;12:7483. doi: 10.1038/s41598-022-11100-3 (PMC9076856; doi:10.1038/s41598-022-11100-3)
Supplement: Supplementary file 1 — Supplementary Information 1. [file 41598_2022_11100_MOESM1_ESM.docx]

Supplementary file 1

Initially selected low-affinity site:

RPSGRKSSKMQAFRIWDVNQKTFYLRNNQLVAGYLQGPNVNLEEKIDVVPIEPHALFLGIHGGKMCLSCVKSGDETRLQLEAVNITDLSENRKQDKRFAFIRSDSGPTTSFESAACPGWFLCTAMEADQPVSLTNMPDEGVMVTKFYFQEDE

Selected T-IL-1RA model:

RPSGRKSSKMQAFRIWDVNQKTFYLRNNQLVAGYLQGPNVNLEEKIDVVPIEPHALFLGIHGGKMCLSCVKSGDETRLQLEAVNITDLSENRKQDKRFAFIRSDSGPTTSFESAACPGWFLCTAMEADQPVSLTNMPDEGVMVTKFYFQEDE

Supplementary file 1: Selected low-affinity sites of IL-1RA highlighted in red. Modified sequence of T-IL-1RA model with the deleted sites highlighted in red.
