# Supplementary material for: Optimization of IL-1RA structure to achieve a smaller protein with a higher affinity to its receptor
Source: Sci Rep. 2022 May 6;12:7483. doi: 10.1038/s41598-022-11100-3 (PMC9076856; doi:10.1038/s41598-022-11100-3)
Supplement: Supplementary file 2 — Supplementary Information 2. [file 41598_2022_11100_MOESM2_ESM.docx]

Supplementary file 2

1

RPSGRKSSKMQAFRIWDVNQKTFYLRNNQLVAGYLQGPNVNLEEKIDVVPIEPHALFLGIHGGKMCLSCVKSGDETRLQLEAVNITDLSENRKQDKRFAFIRSDSGPTTSFESAACPGWFLCTAMEADQPVSLTNMPDEGVMVTKFYFQEDE

2

RPSGRKSSKMQAFRIWDVNQKTFYLRNNQLVAGYLQGPNVNLEEKIDVVPIEPHALFLGIHGGKMCLSCVKSGDETRLQLEAVNITDLSENRKQDKRFAFIRSDSGPTTSFESAACPGWFLCTAMEADQPVSLTNMPDEGVMVTKFYFQEDE

3

RPSGRKSSKMQAFRIWDVNQKTFYLRNNQLVAGYLQGPNVNLEEKIDVVPIEPHALFLGIHGGKMCLSCVKSGDETRLQLEAVNITDLSENRKQDKRFAFIRSDSGPTTSFESAACPGWFLCTAMEADQPVSLTNMPDEGVMVTKFYFQEDE

4

RPSGRKSSKMQAFRIWDVNQKTFYLRNNQLVAGYLQGPNVNLEEKIDVVPIEPHALFLGIHGGKMCLSCVKSGDETRLQLEAVNITDLSENRKQDKRFAFIRSDSGPTTSFESAACPGWFLCTAMEADQPVSLTNMPDEGVMVTKFYFQEDE

5

RPSGRKSSKMQAFRIWDVNQKTFYLRNNQLVAGYLQGPNVNLEEKIDVVPIEPHALFLGIHGGKMCLSCVKSGDETRLQLEAVNITDLSENRKQDKRFAFIRSDSGPTTSFESAACPGWFLCTAMEADQPVSLTNMPDEGVMVTKFYFQEDE

6

RPSGRKSSKMQAFRIWDVNQKTFYLRNNQLVAGYLQGPNVNLEEKIDVVPIEPHALFLGIHGGKMCLSCVKSGDETRLQLEAVNITDLSENRKQDKRFAFIRSDSGPTTSFESAACPGWFLCTAMEADQPVSLTNMPDEGVMVTKFYFQEDE

7

RPSGRKSSKMQAFRIWDVNQKTFYLRNNQLVAGYLQGPNVNLEEKIDVVPIEPHALFLGIHGGKMCLSCVKSGDETRLQLEAVNITDLSENRKQDKRFAFIRSDSGPTTSFESAACPGWFLCTAMEADQPVSLTNMPDEGVMVTKFYFQEDE

8

RPSGRKSSKMQAFRIWDVNQKTFYLRNNQLVAGYLQGPNVNLEEKIDVVPIEPHALFLGIHGGKMCLSCVKSGDETRLQLEAVNITDLSENRKQDKRFAFIRSDSGPTTSFESAACPGWFLCTAMEADQPVSLTNMPDEGVMVTKFYFQEDE

9

RPSGRKSSKMQAFRIWDVNQKTFYLRNNQLVAGYLQGPNVNLEEKIDVVPIEPHALFLGIHGGKMCLSCVKSGDETRLQLEAVNITDLSENRKQDKRFAFIRSDSGPTTSFESAACPGWFLCTAMEADQPVSLTNMPDEGVMVTKFYFQEDE

10

RPSGRKSSKMQAFRIWDVNQKTFYLRNNQLVAGYLQGPNVNLEEKIDVVPIEPHALFLGIHGGKMCLSCVKSGDETRLQLEAVNITDLSENRKQDKRFAFIRSDSGPTTSFESAACPGWFLCTAMEADQPVSLTNMPDEGVMVTKFYFQEDE

11

RPSGRKSSKMQAFRIWDVNQKTFYLRNNQLVAGYLQGPNVNLEEKIDVVPIEPHALFLGIHGGKMCLSCVKSGDETRLQLEAVNITDLSENRKQDKRFAFIRSDSGPTTSFESAACPGWFLCTAMEADQPVSLTNMPDEGVMVTKFYFQEDE

12

RPSGRKSSKMQAFRIWDVNQKTFYLRNNQLVAGYLQGPNVNLEEKIDVVPIEPHALFLGIHGGKMCLSCVKSGDETRLQLEAVNITDLSENRKQDKRFAFIRSDSGPTTSFESAACPGWFLCTAMEADQPVSLTNMPDEGVMVTKFYFQEDE

13

RPSGRKSSKMQAFRIWDVNQKTFYLRNNQLVAGYLQGPNVNLEEKIDVVPIEPHALFLGIHGGKMCLSCVKSGDETRLQLEAVNITDLSENRKQDKRFAFIRSDSGPTTSFESAACPGWFLCTAMEADQPVSLTNMPDEGVMVTKFYFQEDE

14

RPSGRKSSKMQAFRIWDVNQKTFYLRNNQLVAGYLQGPNVNLEEKIDVVPIEPHALFLGIHGGKMCLSCVKSGDETRLQLEAVNITDLSENRKQDKRFAFIRSDSGPTTSFESAACPGWFLCTAMEADQPVSLTNMPDEGVMVTKFYFQEDE

15

RPSGRKSSKMQAFRIWDVNQKTFYLRNNQLVAGYLQGPNVNLEEKIDVVPIEPHALFLGIHGGKMCLSCVKSGDETRLQLEAVNITDLSENRKQDKRFAFIRSDSGPTTSFESAACPGWFLCTAMEADQPVSLTNMPDEGVMVTKFYFQEDE

16

RPSGRKSSKMQAFRIWDVNQKTFYLRNNQLVAGYLQGPNVNLEEKIDVVPIEPHALFLGIHGGKMCLSCVKSGDETRLQLEAVNITDLSENRKQDKRFAFIRSDSGPTTSFESAACPGWFLCTAMEADQPVSLTNMPDEGVMVTKFYFQEDE

17

RPSGRKSSKMQAFRIWDVNQKTFYLRNNQLVAGYLQGPNVNLEEKIDVVPIEPHALFLGIHGGKMCLSCVKSGDETRLQLEAVNITDLSENRKQDKRFAFIRSDSGPTTSFESAACPGWFLCTAMEADQPVSLTNMPDEGVMVTKFYFQEDE

18

RPSGRKSSKMQAFRIWDVNQKTFYLRNNQLVAGYLQGPNVNLEEKIDVVPIEPHALFLGIHGGKMCLSCVKSGDETRLQLEAVNITDLSENRKQDKRFAFIRSDSGPTTSFESAACPGWFLCTAMEADQPVSLTNMPDEGVMVTKFYFQEDE

19

RPSGRKSSKMQAFRIWDVNQKTFYLRNNQLVAGYLQGPNVNLEEKIDVVPIEPHALFLGIHGGKMCLSCVKSGDETRLQLEAVNITDLSENRKQDKRFAFIRSDSGPTTSFESAACPGWFLCTAMEADQPVSLTNMPDEGVMVTKFYFQEDE

20

RPSGRKSSKMQAFRIWDVNQKTFYLRNNQLVAGYLQGPNVNLEEKIDVVPIEPHALFLGIHGGKMCLSCVKSGDETRLQLEAVNITDLSENRKQDKRFAFIRSDSGPTTSFESAACPGWFLCTAMEADQPVSLTNMPDEGVMVTKFYFQEDE

21

RPSGRKSSKMQAFRIWDVNQKTFYLRNNQLVAGYLQGPNVNLEEKIDVVPIEPHALFLGIHGGKMCLSCVKSGDETRLQLEAVNITDLSENRKQDKRFAFIRSDSGPTTSFESAACPGWFLCTAMEADQPVSLTNMPDEGVMVTKFYFQEDE

22

RPSGRKSSKMQAFRIWDVNQKTFYLRNNQLVAGYLQGPNVNLEEKIDVVPIEPHALFLGIHGGKMCLSCVKSGDETRLQLEAVNITDLSENRKQDKRFAFIRSDSGPTTSFESAACPGWFLCTAMEADQPVSLTNMPDEGVMVTKFYFQEDE

23

RPSGRKSSKMQAFRIWDVNQKTFYLRNNQLVAGYLQGPNVNLEEKIDVVPIEPHALFLGIHGGKMCLSCVKSGDETRLQLEAVNITDLSENRKQDKRFAFIRSDSGPTTSFESAACPGWFLCTAMEADQPVSLTNMPDEGVMVTKFYFQEDE

24

RPSGRKSSKMQAFRIWDVNQKTFYLRNNQLVAGYLQGPNVNLEEKIDVVPIEPHALFLGIHGGKMCLSCVKSGDETRLQLEAVNITDLSENRKQDKRFAFIRSDSGPTTSFESAACPGWFLCTAMEADQPVSLTNMPDEGVMVTKFYFQEDE

25

RPSGRKSSKMQAFRIWDVNQKTFYLRNNQLVAGYLQGPNVNLEEKIDVVPIEPHALFLGIHGGKMCLSCVKSGDETRLQLEAVNITDLSENRKQDKRFAFIRSDSGPTTSFESAACPGWFLCTAMEADQPVSLTNMPDEGVMVTKFYFQEDE

26

RPSGRKSSKMQAFRIWDVNQKTFYLRNNQLVAGYLQGPNVNLEEKIDVVPIEPHALFLGIHGGKMCLSCVKSGDETRLQLEAVNITDLSENRKQDKRFAFIRSDSGPTTSFESAACPGWFLCTAMEADQPVSLTNMPDEGVMVTKFYFQEDE

27 RPSGRKSSKMQAFRIWDVNQKTFYLRNNQLVAGYLQGPNVNLEEKIDVVPIEPHALFLGIHGGKMCLSCVKSGDETRLQLEAVNITDLSENRKQDKRFAFIRSDSGPTTSFESAACPGWFLCTAMEADQPVSLTNMPDEGVMVTKFYFQEDE

28

RPSGRKSSKMQAFRIWDVNQKTFYLRNNQLVAGYLQGPNVNLEEKIDVVPIEPHALFLGIHGGKMCLSCVKSGDETRLQLEAVNITDLSENRKQDKRFAFIRSDSGPTTSFESAACPGWFLCTAMEADQPVSLTNMPDEGVMVTKFYFQEDE

29

RPSGRKSSKMQAFRIWDVNQKTFYLRNNQLVAGYLQGPNVNLEEKIDVVPIEPHALFLGIHGGKMCLSCVKSGDETRLQLEAVNITDLSENRKQDKRFAFIRSDSGPTTSFESAACPGWFLCTAMEADQPVSLTNMPDEGVMVTKFYFQEDE

30

RPSGRKSSKMQAFRIWDVNQKTFYLRNNQLVAGYLQGPNVNLEEKIDVVPIEPHALFLGIHGGKMCLSCVKSGDETRLQLEAVNITDLSENRKQDKRFAFIRSDSGPTTSFESAACPGWFLCTAMEADQPVSLTNMPDEGVMVTKFYFQEDE

31

RPSGRKSSKMQAFRIWDVNQKTFYLRNNQLVAGYLQGPNVNLEEKIDVVPIEPHALFLGIHGGKMCLSCVKSGDETRLQLEAVNITDLSENRKQDKRFAFIRSDSGPTTSFESAACPGWFLCTAMEADQPVSLTNMPDEGVMVTKFYFQEDE

32

RPSGRKSSKMQAFRIWDVNQKTFYLRNNQLVAGYLQGPNVNLEEKIDVVPIEPHALFLGIHGGKMCLSCVKSGDETRLQLEAVNITDLSENRKQDKRFAFIRSDSGPTTSFESAACPGWFLCTAMEADQPVSLTNMPDEGVMVTKFYFQEDE

33

RPSGRKSSKMQAFRIWDVNQKTFYLRNNQLVAGYLQGPNVNLEEKIDVVPIEPHALFLGIHGGKMCLSCVKSGDETRLQLEAVNITDLSENRKQDKRFAFIRSDSGPTTSFESAACPGWFLCTAMEADQPVSLTNMPDEGVMVTKFYFQEDE

34

RPSGRKSSKMQAFRIWDVNQKTFYLRNNQLVAGYLQGPNVNLEEKIDVVPIEPHALFLGIHGGKMCLSCVKSGDETRLQLEAVNITDLSENRKQDKRFAFIRSDSGPTTSFESAACPGWFLCTAMEADQPVSLTNMPDEGVMVTKFYFQEDE

35

RPSGRKSSKMQAFRIWDVNQKTFYLRNNQLVAGYLQGPNVNLEEKIDVVPIEPHALFLGIHGGKMCLSCVKSGDETRLQLEAVNITDLSENRKQDKRFAFIRSDSGPTTSFESAACPGWFLCTAMEADQPVSLTNMPDEGVMVTKFYFQEDE

36

RPSGRKSSKMQAFRIWDVNQKTFYLRNNQLVAGYLQGPNVNLEEKIDVVPIEPHALFLGIHGGKMCLSCVKSGDETRLQLEAVNITDLSENRKQDKRFAFIRSDSGPTTSFESAACPGWFLCTAMEADQPVSLTNMPDEGVMVTKFYFQEDE

37

RPSGRKSSKMQAFRIWDVNQKTFYLRNNQLVAGYLQGPNVNLEEKIDVVPIEPHALFLGIHGGKMCLSCVKSGDETRLQLEAVNITDLSENRKQDKRFAFIRSDSGPTTSFESAACPGWFLCTAMEADQPVSLTNMPDEGVMVTKFYFQEDE

38

RPSGRKSSKMQAFRIWDVNQKTFYLRNNQLVAGYLQGPNVNLEEKIDVVPIEPHALFLGIHGGKMCLSCVKSGDETRLQLEAVNITDLSENRKQDKRFAFIRSDSGPTTSFESAACPGWFLCTAMEADQPVSLTNMPDEGVMVTKFYFQEDE

39

RPSGRKSSKMQAFRIWDVNQKTFYLRNNQLVAGYLQGPNVNLEEKIDVVPIEPHALFLGIHGGKMCLSCVKSGDETRLQLEAVNITDLSENRKQDKRFAFIRSDSGPTTSFESAACPGWFLCTAMEADQPVSLTNMPDEGVMVTKFYFQEDE

40

RPSGRKSSKMQAFRIWDVNQKTFYLRNNQLVAGYLQGPNVNLEEKIDVVPIEPHALFLGIHGGKMCLSCVKSGDETRLQLEAVNITDLSENRKQDKRFAFIRSDSGPTTSFESAACPGWFLCTAMEADQPVSLTNMPDEGVMVTKFYFQEDE

41

RPSGRKSSKMQAFRIWDVNQKTFYLRNNQLVAGYLQGPNVNLEEKIDVVPIEPHALFLGIHGGKMCLSCVKSGDETRLQLEAVNITDLSENRKQDKRFAFIRSDSGPTTSFESAACPGWFLCTAMEADQPVSLTNMPDEGVMVTKFYFQEDE

42

RPSGRKSSKMQAFRIWDVNQKTFYLRNNQLVAGYLQGPNVNLEEKIDVVPIEPHALFLGIHGGKMCLSCVKSGDETRLQLEAVNITDLSENRKQDKRFAFIRSDSGPTTSFESAACPGWFLCTAMEADQPVSLTNMPDEGVMVTKFYFQEDE

43

RPSGRKSSKMQAFRIWDVNQKTFYLRNNQLVAGYLQGPNVNLEEKIDVVPIEPHALFLGIHGGKMCLSCVKSGDETRLQLEAVNITDLSENRKQDKRFAFIRSDSGPTTSFESAACPGWFLCTAMEADQPVSLTNMPDEGVMVTKFYFQEDE

44

RPSGRKSSKMQAFRIWDVNQKTFYLRNNQLVAGYLQGPNVNLEEKIDVVPIEPHALFLGIHGGKMCLSCVKSGDETRLQLEAVNITDLSENRKQDKRFAFIRSDSGPTTSFESAACPGWFLCTAMEADQPVSLTNMPDEGVMVTKFYFQEDE

45

RPSGRKSSKMQAFRIWDVNQKTFYLRNNQLVAGYLQGPNVNLEEKIDVVPIEPHALFLGIHGGKMCLSCVKSGDETRLQLEAVNITDLSENRKQDKRFAFIRSDSGPTTSFESAACPGWFLCTAMEADQPVSLTNMPDEGVMVTKFYFQEDE

46

RPSGRKSSKMQAFRIWDVNQKTFYLRNNQLVAGYLQGPNVNLEEKIDVVPIEPHALFLGIHGGKMCLSCVKSGDETRLQLEAVNITDLSENRKQDKRFAFIRSDSGPTTSFESAACPGWFLCTAMEADQPVSLTNMPDEGVMVTKFYFQEDE

47

RPSGRKSSKMQAFRIWDVNQKTFYLRNNQLVAGYLQGPNVNLEEKIDVVPIEPHALFLGIHGGKMCLSCVKSGDETRLQLEAVNITDLSENRKQDKRFAFIRSDSGPTTSFESAACPGWFLCTAMEADQPVSLTNMPDEGVMVTKFYFQEDE

48

RPSGRKSSKMQAFRIWDVNQKTFYLRNNQLVAGYLQGPNVNLEEKIDVVPIEPHALFLGIHGGKMCLSCVKSGDETRLQLEAVNITDLSENRKQDKRFAFIRSDSGPTTSFESAACPGWFLCTAMEADQPVSLTNMPDEGVMVTKFYFQEDE

49

RPSGRKSSKMQAFRIWDVNQKTFYLRNNQLVAGYLQGPNVNLEEKIDVVPIEPHALFLGIHGGKMCLSCVKSGDETRLQLEAVNITDLSENRKQDKRFAFIRSDSGPTTSFESAACPGWFLCTAMEADQPVSLTNMPDEGVMVTKFYFQEDE

50

RPSGRKSSKMQAFRIWDVNQKTFYLRNNQLVAGYLQGPNVNLEEKIDVVPIEPHALFLGIHGGKMCLSCVKSGDETRLQLEAVNITDLSENRKQDKRFAFIRSDSGPTTSFESAACPGWFLCTAMEADQPVSLTNMPDEGVMVTKFYFQEDE

51

RPSGRKSSKMQAFRIWDVNQKTFYLRNNQLVAGYLQGPNVNLEEKIDVVPIEPHALFLGIHGGKMCLSCVKSGDETRLQLEAVNITDLSENRKQDKRFAFIRSDSGPTTSFESAACPGWFLCTAMEADQPVSLTNMPDEGVMVTKFYFQEDE

52

RPSGRKSSKMQAFRIWDVNQKTFYLRNNQLVAGYLQGPNVNLEEKIDVVPIEPHALFLGIHGGKMCLSCVKSGDETRLQLEAVNITDLSENRKQDKRFAFIRSDSGPTTSFESAACPGWFLCTAMEADQPVSLTNMPDEGVMVTKFYFQEDE

53

RPSGRKSSKMQAFRIWDVNQKTFYLRNNQLVAGYLQGPNVNLEEKIDVVPIEPHALFLGIHGGKMCLSCVKSGDETRLQLEAVNITDLSENRKQDKRFAFIRSDSGPTTSFESAACPGWFLCTAMEADQPVSLTNMPDEGVMVTKFYFQEDE

54

RPSGRKSSKMQAFRIWDVNQKTFYLRNNQLVAGYLQGPNVNLEEKIDVVPIEPHALFLGIHGGKMCLSCVKSGDETRLQLEAVNITDLSENRKQDKRFAFIRSDSGPTTSFESAACPGWFLCTAMEADQPVSLTNMPDEGVMVTKFYFQEDE

55

RPSGRKSSKMQAFRIWDVNQKTFYLRNNQLVAGYLQGPNVNLEEKIDVVPIEPHALFLGIHGGKMCLSCVKSGDETRLQLEAVNITDLSENRKQDKRFAFIRSDSGPTTSFESAACPGWFLCTAMEADQPVSLTNMPDEGVMVTKFYFQEDE

57

RPSGRKSSKMQAFRIWDVNQKTFYLRNNQLVAGYLQGPNVNLEEKIDVVPIEPHALFLGIHGGKMCLSCVKSGDETRLQLEAVNITDLSENRKQDKRFAFIRSDSGPTTSFESAACPGWFLCTAMEADQPVSLTNMPDEGVMVTKFYFQEDE

58

RPSGRKSSKMQAFRIWDVNQKTFYLRNNQLVAGYLQGPNVNLEEKIDVVPIEPHALFLGIHGGKMCLSCVKSGDETRLQLEAVNITDLSENRKQDKRFAFIRSDSGPTTSFESAACPGWFLCTAMEADQPVSLTNMPDEGVMVTKFYFQEDE

59

RPSGRKSSKMQAFRIWDVNQKTFYLRNNQLVAGYLQGPNVNLEEKIDVVPIEPHALFLGIHGGKMCLSCVKSGDETRLQLEAVNITDLSENRKQDKRFAFIRSDSGPTTSFESAACPGWFLCTAMEADQPVSLTNMPDEGVMVTKFYFQEDE

60

RPSGRKSSKMQAFRIWDVNQKTFYLRNNQLVAGYLQGPNVNLEEKIDVVPIEPHALFLGIHGGKMCLSCVKSGDETRLQLEAVNITDLSENRKQDKRFAFIRSDSGPTTSFESAACPGWFLCTAMEADQPVSLTNMPDEGVMVTKFYFQEDE

61

RPSGRKSSKMQAFRIWDVNQKTFYLRNNQLVAGYLQGPNVNLEEKIDVVPIEPHALFLGIHGGKMCLSCVKSGDETRLQLEAVNITDLSENRKQDKRFAFIRSDSGPTTSFESAACPGWFLCTAMEADQPVSLTNMPDEGVMVTKFYFQEDE

62

RPSGRKSSKMQAFRIWDVNQKTFYLRNNQLVAGYLQGPNVNLEEKIDVVPIEPHALFLGIHGGKMCLSCVKSGDETRLQLEAVNITDLSENRKQDKRFAFIRSDSGPTTSFESAACPGWFLCTAMEADQPVSLTNMPDEGVMVTKFYFQEDE

63

RPSGRKSSKMQAFRIWDVNQKTFYLRNNQLVAGYLQGPNVNLEEKIDVVPIEPHALFLGIHGGKMCLSCVKSGDETRLQLEAVNITDLSENRKQDKRFAFIRSDSGPTTSFESAACPGWFLCTAMEADQPVSLTNMPDEGVMVTKFYFQEDE

64

RPSGRKSSKMQAFRIWDVNQKTFYLRNNQLVAGYLQGPNVNLEEKIDVVPIEPHALFLGIHGGKMCLSCVKSGDETRLQLEAVNITDLSENRKQDKRFAFIRSDSGPTTSFESAACPGWFLCTAMEADQPVSLTNMPDEGVMVTKFYFQEDE

65

RPSGRKSSKMQAFRIWDVNQKTFYLRNNQLVAGYLQGPNVNLEEKIDVVPIEPHALFLGIHGGKMCLSCVKSGDETRLQLEAVNITDLSENRKQDKRFAFIRSDSGPTTSFESAACPGWFLCTAMEADQPVSLTNMPDEGVMVTKFYFQEDE

66

RPSGRKSSKMQAFRIWDVNQKTFYLRNNQLVAGYLQGPNVNLEEKIDVVPIEPHALFLGIHGGKMCLSCVKSGDETRLQLEAVNITDLSENRKQDKRFAFIRSDSGPTTSFESAACPGWFLCTAMEADQPVSLTNMPDEGVMVTKFYFQEDE

67

RPSGRKSSKMQAFRIWDVNQKTFYLRNNQLVAGYLQGPNVNLEEKIDVVPIEPHALFLGIHGGKMCLSCVKSGDETRLQLEAVNITDLSENRKQDKRFAFIRSDSGPTTSFESAACPGWFLCTAMEADQPVSLTNMPDEGVMVTKFYFQEDE

68

RPSGRKSSKMQAFRIWDVNQKTFYLRNNQLVAGYLQGPNVNLEEKIDVVPIEPHALFLGIHGGKMCLSCVKSGDETRLQLEAVNITDLSENRKQDKRFAFIRSDSGPTTSFESAACPGWFLCTAMEADQPVSLTNMPDEGVMVTKFYFQEDE

69

RPSGRKSSKMQAFRIWDVNQKTFYLRNNQLVAGYLQGPNVNLEEKIDVVPIEPHALFLGIHGGKMCLSCVKSGDETRLQLEAVNITDLSENRKQDKRFAFIRSDSGPTTSFESAACPGWFLCTAMEADQPVSLTNMPDEGVMVTKFYFQEDE

70

RPSGRKSSKMQAFRIWDVNQKTFYLRNNQLVAGYLQGPNVNLEEKIDVVPIEPHALFLGIHGGKMCLSCVKSGDETRLQLEAVNITDLSENRKQDKRFAFIRSDSGPTTSFESAACPGWFLCTAMEADQPVSLTNMPDEGVMVTKFYFQEDE

71

RPSGRKSSKMQAFRIWDVNQKTFYLRNNQLVAGYLQGPNVNLEEKIDVVPIEPHALFLGIHGGKMCLSCVKSGDETRLQLEAVNITDLSENRKQDKRFAFIRSDSGPTTSFESAACPGWFLCTAMEADQPVSLTNMPDEGVMVTKFYFQEDE

72

RPSGRKSSKMQAFRIWDVNQKTFYLRNNQLVAGYLQGPNVNLEEKIDVVPIEPHALFLGIHGGKMCLSCVKSGDETRLQLEAVNITDLSENRKQDKRFAFIRSDSGPTTSFESAACPGWFLCTAMEADQPVSLTNMPDEGVMVTKFYFQEDE

73

RPSGRKSSKMQAFRIWDVNQKTFYLRNNQLVAGYLQGPNVNLEEKIDVVPIEPHALFLGIHGGKMCLSCVKSGDETRLQLEAVNITDLSENRKQDKRFAFIRSDSGPTTSFESAACPGWFLCTAMEADQPVSLTNMPDEGVMVTKFYFQEDE

74

RPSGRKSSKMQAFRIWDVNQKTFYLRNNQLVAGYLQGPNVNLEEKIDVVPIEPHALFLGIHGGKMCLSCVKSGDETRLQLEAVNITDLSENRKQDKRFAFIRSDSGPTTSFESAACPGWFLCTAMEADQPVSLTNMPDEGVMVTKFYFQEDE

75

RPSGRKSSKMQAFRIWDVNQKTFYLRNNQLVAGYLQGPNVNLEEKIDVVPIEPHALFLGIHGGKMCLSCVKSGDETRLQLEAVNITDLSENRKQDKRFAFIRSDSGPTTSFESAACPGWFLCTAMEADQPVSLTNMPDEGVMVTKFYFQEDE

76

RPSGRKSSKMQAFRIWDVNQKTFYLRNNQLVAGYLQGPNVNLEEKIDVVPIEPHALFLGIHGGKMCLSCVKSGDETRLQLEAVNITDLSENRKQDKRFAFIRSDSGPTTSFESAACPGWFLCTAMEADQPVSLTNMPDEGVMVTKFYFQEDE

77

RPSGRKSSKMQAFRIWDVNQKTFYLRNNQLVAGYLQGPNVNLEEKIDVVPIEPHALFLGIHGGKMCLSCVKSGDETRLQLEAVNITDLSENRKQDKRFAFIRSDSGPTTSFESAACPGWFLCTAMEADQPVSLTNMPDEGVMVTKFYFQEDE

78

RPSGRKSSKMQAFRIWDVNQKTFYLRNNQLVAGYLQGPNVNLEEKIDVVPIEPHALFLGIHGGKMCLSCVKSGDETRLQLEAVNITDLSENRKQDKRFAFIRSDSGPTTSFESAACPGWFLCTAMEADQPVSLTNMPDEGVMVTKFYFQEDE

79

RPSGRKSSKMQAFRIWDVNQKTFYLRNNQLVAGYLQGPNVNLEEKIDVVPIEPHALFLGIHGGKMCLSCVKSGDETRLQLEAVNITDLSENRKQDKRFAFIRSDSGPTTSFESAACPGWFLCTAMEADQPVSLTNMPDEGVMVTKFYFQEDE

80

RPSGRKSSKMQAFRIWDVNQKTFYLRNNQLVAGYLQGPNVNLEEKIDVVPIEPHALFLGIHGGKMCLSCVKSGDETRLQLEAVNITDLSENRKQDKRFAFIRSDSGPTTSFESAACPGWFLCTAMEADQPVSLTNMPDEGVMVTKFYFQEDE

81

RPSGRKSSKMQAFRIWDVNQKTFYLRNNQLVAGYLQGPNVNLEEKIDVVPIEPHALFLGIHGGKMCLSCVKSGDETRLQLEAVNITDLSENRKQDKRFAFIRSDSGPTTSFESAACPGWFLCTAMEADQPVSLTNMPDEGVMVTKFYFQEDE

82

RPSGRKSSKMQAFRIWDVNQKTFYLRNNQLVAGYLQGPNVNLEEKIDVVPIEPHALFLGIHGGKMCLSCVKSGDETRLQLEAVNITDLSENRKQDKRFAFIRSDSGPTTSFESAACPGWFLCTAMEADQPVSLTNMPDEGVMVTKFYFQEDE

83

RPSGRKSSKMQAFRIWDVNQKTFYLRNNQLVAGYLQGPNVNLEEKIDVVPIEPHALFLGIHGGKMCLSCVKSGDETRLQLEAVNITDLSENRKQDKRFAFIRSDSGPTTSFESAACPGWFLCTAMEADQPVSLTNMPDEGVMVTKFYFQEDE

84

RPSGRKSSKMQAFRIWDVNQKTFYLRNNQLVAGYLQGPNVNLEEKIDVVPIEPHALFLGIHGGKMCLSCVKSGDETRLQLEAVNITDLSENRKQDKRFAFIRSDSGPTTSFESAACPGWFLCTAMEADQPVSLTNMPDEGVMVTKFYFQEDE

85

RPSGRKSSKMQAFRIWDVNQKTFYLRNNQLVAGYLQGPNVNLEEKIDVVPIEPHALFLGIHGGKMCLSCVKSGDETRLQLEAVNITDLSENRKQDKRFAFIRSDSGPTTSFESAACPGWFLCTAMEADQPVSLTNMPDEGVMVTKFYFQEDE

86

RPSGRKSSKMQAFRIWDVNQKTFYLRNNQLVAGYLQGPNVNLEEKIDVVPIEPHALFLGIHGGKMCLSCVKSGDETRLQLEAVNITDLSENRKQDKRFAFIRSDSGPTTSFESAACPGWFLCTAMEADQPVSLTNMPDEGVMVTKFYFQEDE

87

RPSGRKSSKMQAFRIWDVNQKTFYLRNNQLVAGYLQGPNVNLEEKIDVVPIEPHALFLGIHGGKMCLSCVKSGDETRLQLEAVNITDLSENRKQDKRFAFIRSDSGPTTSFESAACPGWFLCTAMEADQPVSLTNMPDEGVMVTKFYFQEDE

88

RPSGRKSSKMQAFRIWDVNQKTFYLRNNQLVAGYLQGPNVNLEEKIDVVPIEPHALFLGIHGGKMCLSCVKSGDETRLQLEAVNITDLSENRKQDKRFAFIRSDSGPTTSFESAACPGWFLCTAMEADQPVSLTNMPDEGVMVTKFYFQEDE

89

RPSGRKSSKMQAFRIWDVNQKTFYLRNNQLVAGYLQGPNVNLEEKIDVVPIEPHALFLGIHGGKMCLSCVKSGDETRLQLEAVNITDLSENRKQDKRFAFIRSDSGPTTSFESAACPGWFLCTAMEADQPVSLTNMPDEGVMVTKFYFQEDE

90

RPSGRKSSKMQAFRIWDVNQKTFYLRNNQLVAGYLQGPNVNLEEKIDVVPIEPHALFLGIHGGKMCLSCVKSGDETRLQLEAVNITDLSENRKQDKRFAFIRSDSGPTTSFESAACPGWFLCTAMEADQPVSLTNMPDEGVMVTKFYFQEDE

91

RPSGRKSSKMQAFRIWDVNQKTFYLRNNQLVAGYLQGPNVNLEEKIDVVPIEPHALFLGIHGGKMCLSCVKSGDETRLQLEAVNITDLSENRKQDKRFAFIRSDSGPTTSFESAACPGWFLCTAMEADQPVSLTNMPDEGVMVTKFYFQEDE

92

RPSGRKSSKMQAFRIWDVNQKTFYLRNNQLVAGYLQGPNVNLEEKIDVVPIEPHALFLGIHGGKMCLSCVKSGDETRLQLEAVNITDLSENRKQDKRFAFIRSDSGPTTSFESAACPGWFLCTAMEADQPVSLTNMPDEGVMVTKFYFQEDE

93

RPSGRKSSKMQAFRIWDVNQKTFYLRNNQLVAGYLQGPNVNLEEKIDVVPIEPHALFLGIHGGKMCLSCVKSGDETRLQLEAVNITDLSENRKQDKRFAFIRSDSGPTTSFESAACPGWFLCTAMEADQPVSLTNMPDEGVMVTKFYFQEDE

94

RPSGRKSSKMQAFRIWDVNQKTFYLRNNQLVAGYLQGPNVNLEEKIDVVPIEPHALFLGIHGGKMCLSCVKSGDETRLQLEAVNITDLSENRKQDKRFAFIRSDSGPTTSFESAACPGWFLCTAMEADQPVSLTNMPDEGVMVTKFYFQEDE

RPSGRKSSKMQAFRIWDVNQKTFYLRNNQLVAGYLQGPNVNLEEKIDVVPIEPHALFLGIHGGKMCLIRSDSGPTTSFESAACPGWFLCTAMEADQPVSLTNMPDEGVMVTKFYFQEDE

95

RPSGRKSSKMQAFRIWDVNQKTFYLRNNQLVAGYLQGPNVNLEEKIDVVPIEPHALFLGIHGGKMCLSCVKSGDETRLQLEAVNITDLSENRKQDKRFAFIRSDSGPTTSFESAACPGWFLCTAMEADQPVSLTNMPDEGVMVTKFYFQEDE

96

RPSGRKSSKMQAFRIWDVNQKTFYLRNNQLVAGYLQGPNVNLEEKIDVVPIEPHALFLGIHGGKMCLSCVKSGDETRLQLEAVNITDLSENRKQDKRFAFIRSDSGPTTSFESAACPGWFLCTAMEADQPVSLTNMPDEGVMVTKFYFQEDE

97

RPSGRKSSKMQAFRIWDVNQKTFYLRNNQLVAGYLQGPNVNLEEKIDVVPIEPHALFLGIHGGKMCLSCVKSGDETRLQLEAVNITDLSENRKQDKRFAFIRSDSGPTTSFESAACPGWFLCTAMEADQPVSLTNMPDEGVMVTKFYFQEDE

98

RPSGRKSSKMQAFRIWDVNQKTFYLRNNQLVAGYLQGPNVNLEEKIDVVPIEPHALFLGIHGGKMCLSCVKSGDETRLQLEAVNITDLSENRKQDKRFAFIRSDSGPTTSFESAACPGWFLCTAMEADQPVSLTNMPDEGVMVTKFYFQEDE

99

RPSGRKSSKMQAFRIWDVNQKTFYLRNNQLVAGYLQGPNVNLEEKIDVVPIEPHALFLGIHGGKMCLSCVKSGDETRLQLEAVNITDLSENRKQDKRFAFIRSDSGPTTSFESAACPGWFLCTAMEADQPVSLTNMPDEGVMVTKFYFQEDE

100

RPSGRKSSKMQAFRIWDVNQKTFYLRNNQLVAGYLQGPNVNLEEKIDVVPIEPHALFLGIHGGKMCLSCVKSGDETRLQLEAVNITDLSENRKQDKRFAFIRSDSGPTTSFESAACPGWFLCTAMEADQPVSLTNMPDEGVMVTKFYFQEDE

Supplementary file 2: Selected 100 models for docking with the deleted sites highlighted in red.
